# Are Patients with Polycystic Ovarian Syndrome Ideal Candidates for Oocyte Donation?

**DOI:** 10.1155/2016/5701609

**Published:** 2016-04-12

**Authors:** George Queiroz Vaz, Alessandra Viviane Evangelista, Cassio Alessandro Paganoti Sartorio, Maria Cecilia Almeida Cardoso, Maria Cecilia Erthal, Paulo Gallo, Marco Aurelio Pinho Oliveira

**Affiliations:** ^1^Department of Gynecology, Rio de Janeiro State University, 20551-030 Rio de Janeiro, RJ, Brazil; ^2^Vida Fertility Center, 22793-080 Rio de Janeiro, RJ, Brazil

## Abstract

*Background.* The use of donated oocytes for in vitro fertilization treatment in patients with ovarian failure is universally recognized. But would patients with polycystic ovarian syndrome (PCOS) be a good choice for egg donation programs?* Objective.* Comparing the pregnancy rates of egg receptors from donor patients diagnosed with PCOS to receptors from donors without PCOS.* Design.* Retrospective cohort study.* Methods.* A total of 234 patients who had undergone egg reception program were separated into two groups: Group I, receptors from PCOS donors (*n* = 36); Group II, receptors from donors without PCOS (*n* = 198). Medical records were reviewed and the fertilization, implantation, and pregnancy rates were calculated.* Results.* PCOS patients had an average of 3.23 more oocytes retrieved, but there were no differences in the number of mature oocytes that were used for donation between the groups. We also observed that the number of transferred embryos was also not significantly different, as well as the fertilization and implantation rates. The clinical pregnancy rates were not significantly different: 28% and 26% in Group I and Group II, respectively.* Conclusions.* Women with PCOS should not be excluded from egg donation programs.

## 1. Introduction

The use of donor eggs in the treatment for infertile women, particularly in cases of ovarian failure, genetic diseases, and failed in vitro fertilization (IVF) after oocyte recovery, has considerably increased in recent decades. The success of oocyte donation may be influenced by various factors, including the donor's age, quality and number of embryos transferred, and recipient's age and endometrial receptivity; donor age is undoubtedly the most important factor contributing to treatment success [1].

There are different practices for obtaining donor oocytes. They can be obtained from the following sources: (a) nonpatient donors, which include volunteer (those who receive no financial compensation) and known (donation to a known recipient) donors; (b) commercial donors (those who receive financial compensation); and (c) patient donors (those scheduled to undergo IVF). Generally, these women make an agreement with the fertility clinic to donate some of their oocytes for treatment purposes in order to receive infertility treatment at a discounted rate; more rarely, they are patients who voluntarily donate excess oocytes from their own treatments [2]. In Brazil, the law allows only donation of oocytes from patient donors. In addition to concerns pertaining to donor age, Brazilian physicians must consider the infertility factor that led the donor to receive IVF and must consider whether this factor will influence the pregnancy result in the recipient [3].

Polycystic ovarian syndrome (PCOS) is an endocrine disorder and is commonly associated with infertility; it is most commonly seen in women of reproductive age, and its prevalence is estimated to vary from 6% to 21% [4, 5].

The use of ovulation induction drugs is the first alternative for the treatment of infertile women with PCOS; however, the reproductive technologies are being increasingly applied. The IVF became a frequent alternative to ovarian induction [6], making it possible that more patients with PCOS are able to participate in oocyte donation programs as donor patients.

Patients with PCOS who undergo IVF present greater oocyte production, require less gonadotropin, and are more likely to experience ovarian hyperstimulation syndrome (OHSS) and miscarriages. However, the rates of pregnancy are equivalent to those in patients with other causes of infertility [6–9]. Heijnen et al. [6] included 9 studies in a meta-analysis that compared reproductive IVF outcomes in patients with and without PCOS. They found no statistical difference between pregnancy and the live born rates; however, patients with PCOS had a lower oocyte fertilization rate.

Another important concern is the impact on pregnancy rates when patients with PCOS donate their oocytes for IVF. The literature includes only a few studies that evaluate pregnancy rates and miscarriage risk among patients who have received oocytes from those with PCOS [10, 11].

The objective of the present study was to compare the rate of pregnancy in women who have undergone IVF as recipients of oocytes from patients with PCOS and that in women who have undergone IVF as recipients of oocytes from patients with other pathologies; we subsequently determined whether patients with PCOS are an ideal choice for oocyte donation programs.

## 2. Materials and Methods

A retrospective cohort study with secondary data analysis was conducted. The data was obtained from a database and from a review of medical records of oocyte recipients enrolled in an IVF program with donor oocytes at the Vida Fertility Center, a private infertility center in the city and state of Rio de Janeiro, Brazil, between January 2007 and December 2013.

A form was created to collect data from the participants' medical records; this form obtained information on the following variables: recipient and donor age, infertility factor, total number of gonadotropin ampoules used, number of days of stimulation, number of follicles with an average diameter of >14 mm on the day of human chorionic gonadotropin (hCG), number of oocytes retrieved from the donor, number of mature oocytes (metaphase II) that were used in the recipient, number of embryos fertilized on the first day after IVF was used, technique used for IVF (conventional IVF or ICSI), number of embryos transferred, number of embryos frozen, rate of fertilization, rate of implantation, and rate of pregnancy.

This study was approved by the Research Ethics Committee of the Pedro Ernesto University Hospital at Rio de Janeiro State University (UERJ).

A total of 251 patients between 18 and 50 years of age were recipients in an IVF treatment with donated oocytes; 17 patients were excluded due to lack of information, and 234 patients were thereby included.

Ovarian insufficiency was the indication for all recipients. They were divided into two groups: the case group comprised 36 patients who received donor oocytes from women with PCOS who had been diagnosed following the Rotterdam criteria [12] and the control group comprised 198 women who received donor oocytes from women with other infertility diagnoses, excluding PCOS.

### 2.1. Outcome Objectives

The primary objective was to compare the pregnancy rate between the groups. Conception was defined as pregnancy when the presence of a fetal heartbeat was confirmed via transvaginal ultrasound. The secondary objective was to compare the implantation and fertilization rates between the groups. The fertilization rate was defined as the ratio of embryos with two pronuclei (2PN) from recipients on day 1 divided by the number of mature oocytes that were used in the donation. Further, the implantation rate was defined as the number of visible gestational sacs divided by the number of embryos transferred.

### 2.2. IVF

All participating donors underwent conventional controlled ovarian hyperstimulation using the long protocol with gonadotropin-releasing hormone analogs or the short protocol with gonadotropin-releasing hormone-agonist drugs. The gonadotropins used were recombinant follicle-stimulating hormone and/or human menopausal gonadotropin. The stimulation protocol was individualized according to each woman's clinical history, particularly according to chronological age, basal follicle-stimulating hormone levels, and antral follicle count. The protocol was adjusted according to ovarian response, which was evaluated based on ultrasound and serum E2 levels. The recombinant hCG protocol was used to induce follicle maturation when at least two follicles reached an average diameter of 18 mm. Oocyte recovery was performed 34–36 h after hCG injection.

All endometrial preparations from the recipients were performed using an initial dose of 4 mg estradiol/day, reaching up to 6 mg/day, to achieve an endometrium larger than 8 mm in size, as assessed via an ultrasonographic measurement. Progesterone was initiated on the day that the oocytes were removed from the donor, and its administration was continued until the pregnancy test.

### 2.3. Statistical Analyses

Data were analyzed using the Statistical Package for the Social Sciences software (ver. 20). Student's *t*-test was used to compare group averages, and chi-squared test was used to compare pregnancy, fertilization, and implantation rates. Statistical significance was defined as *p* < 0.05 for all comparisons.

## 3. Results

The average recipient age among those who received oocytes from non-PCOS patients was 42 (±5) years; the average recipient age among those who received oocytes from PCOS patients was also 42 (±5) years. Meanwhile, the average age of the donors with PCOS was 30 (±3.51) years; the average age of the donors without an ovary-related pathology was 29 (±3.49) years. There was no difference in the average age between either the recipients or the donors.

When stimulated, patients with PCOS required significantly less gonadotropin in their simulations than the donors without PCOS (1722 IUs ± 738 versus 2089 IUs ± 662). On average, they used 367 fewer IUs of gonadotropins; however, the number of days of stimulation was similar between the groups (9.84 ± 2.95 versus 10.19 ± 3.14). The response to stimulation is presented in Table 1.

Another significant variable was the number of oocytes retrieved from the donor. On average, 3.23 more oocytes were retrieved from patients with PCOS (18.40 ± 8.21 versus 21.63 ± 10.44); however, no differences were found between the groups in terms of the number of mature oocytes (7.91 ± 3.25 versus 7.39 ± 2.92) that could be used for donation.

When the data on the embryos were analyzed, we found that the number of embryos transferred (2.23 ± 0.64 versus 2.65 ± 0.96) and number of embryos frozen (3.39 ± 2.24 versus 3.88 ± 2.18) did not significantly differ between the groups (Table 2).

Clinical pregnancy rates were 28% and 26% (*p* = 0.83) in the PCOS and non-PCOS donor oocyte recipients, respectively. Neither the pregnancy rates nor the fertilization (11% versus 13%) or implantation (69% versus 67%) rates differ significantly, as shown in Table 3.

## 4. Discussion

PCOS affects 6%–21% of women of reproductive age and is an important cause of infertility [4, 5]. An increasing number of women with this syndrome are looking for IVF as an alternative to achieve pregnancy and frequently they are included as oocyte donors.

Thus, there is some concern that because patients with PCOS present an ovary-related pathology, they may be a poor choice for an oocyte donation program. In our study, some aspects of ovarian response in patients with PCOS can actually be regarded as positive, such as the reduced need for gonadotropin and the greater number of oocytes retrieved (as reflected in the findings of our study). This is possibly due to an increased sensitivity to gonadotropins, which is well known and frequently reported in the medical literature [7, 8]; it could also be due to a larger cohort in terms of numbers of follicles [8]. The need for less gonadotropin reduces the costs of ovarian stimulation. However, some additional costs may be needed to treat more patients with OHSS. The severe OHSS has an incidence about 1% of the treatment cycles which often requires hospitalization. It should be noted that these complications can be avoided most of the times following an adequate protocol [13, 14]. In our 36 women with PCOS, there were no cases of severe OHSS.

There are concerns that women with PCOS may produce lower quality oocytes and therefore decrease the oocyte fertilization rate. Niu et al. suggested that oocyte developmental competence is associated with abnormal lipid metabolism and that obese women with PCOS present high concentrations of linoleic acid and palmitoleic acid both in the plasma and in the follicular fluid, which may contribute to the poor pregnancy results of IVF in patients with PCOS [15].

Heijnen et al. [6] did not find statistical difference in the amount of gonadotropins prescribed to patients with and without PCOS. However, they found that PCOS patients had an increased number of oocytes retrieved and an increase in the number of days of stimulation. In the same study, the authors concluded that the PCOS patients presented a decreased oocyte fertilization rate but they had the same pregnancy and live birth rates compared with patients without PCOS.

In our study, we did not observe changes in terms of the number of oocytes retrieved, fertilization rates, or pregnancy rates between women with PCOS and those without ovary-related pathologies. There was also no difference in the number of embryos with 2PN on day 1. These findings are consistent with those reported in the literature.

This result is consistent with the findings reported by Zhong et al. [16], who did not find differences in the number of embryos transferred, clinical pregnancy rates, or implantation rates between women with PCOS and those without PCOS.

Polycystic ovarian morphology is a frequent finding in cases of PCOS and is defined by an excess number of follicles per ovary or by an increase in ovarian volume [17]. The clinical significance of the isolated findings of polycystic ovarian morphology is not well known and there is some evidence that patients with PCOS and polycystic ovarian morphology do not share the same metabolic disorders [18, 19].

The prospective study of Sigala et al., comparing 97 women with ovarian polycystic morphology with 97 women with normal morphology, did not find differences in the oocyte quality, in the fertilization rates, and in the clinic pregnancy rate between the groups [20].

Cho et al. compared 113 women with apparently normal ovaries to 36 women with isolated polycystic ovarian morphology and found that, similar to cases of PCOS, women with polycystic ovarian morphology produced more oocytes and also exhibited increased sensitivity to gonadotropins; even so, the researchers found equivalent rates of fertility and pregnancy between the groups [11].

Although we have a total of 234 patients, we included only 36 oocyte donors with PCOS diagnosis. This small number has the potential to cause a type II error. Other potential limitations include the retrospective analysis of an established database.

Finally, our results suggest that PCOS donors do not differ from donors without ovary-related pathologies in terms of implantation or pregnancy rates.

## 5. Conclusions

PCOS in donors does not seem to affect the pregnancy rates or implantation rate or the numbers of embryos transferred in oocyte donation programs. Women with PCOS should not be excluded from oocyte donation programs. However, strategies that minimize the occurrence of ovarian hyperstimulation syndrome should be adopted.

## Figures and Tables

**Table 1:** Characteristics of ovarian response among patients with and without PCOS (average: standard deviation, SD).

	No PCOS (*n* = 198)	PCOS (*n* = 36)	*p*
Donor age	29 (±3.49 SD)	30 (±3.51 SD)	0.92
Gonadotropins used (total in IU)	2089 (±662 SD)	1722 (±738 SD)	<0.05
Duration of stimulation (days)	9.84 (±2.95 SD)	10.19 (±3.14 SD)	0.51
Follicles > 14 mm	11.08 (±4.82 SD)	12.53 (±4.55 SD)	0.1
Oocytes retrieved (total)	18.40 (±8.21 SD)	21.63 (±10.44 SD)	<0.05

**Table 2:** IVF characteristics of patients receiving oocytes from donors with PCOS and those without PCOS (average: standard deviation, SD).

	No PCOS (*n* = 198)	PCOS (*n* = 36)	*p*
Recipient age	42 (±5 SD)	42 (±5 SD)	0.92
Mature oocytes used in recipient	7.91 (±3.25 SD)	7.39 (±2.92 SD)	0.37
ICSI (%)	90 (178/198)	89 (32/36)	0.85
Day 1 embryos (2PN)^*∗*^	5.21 (±2.85 SD)	5.11 (±2.79 SD)	0.85
Embryos transferred	2.23 (±0.64 SD)	2.65 (±0.96 SD)	0.56
Embryos frozen	3.39 (±2.24 SD)	3.88 (±2.18 SD)	0.43

^*∗*^Two pronuclei.

**Table 3:** Clinical results of patients receiving oocytes from donors with PCOS and those without PCOS.

	No PCOS (*n* = 198)	PCOS (*n* = 36)	*p*
Implantation rate (%)	13 (51/379)	11 (7/61)	0.83
Fertilization rate (%)	67 (1032/1551)	69 (184/266)	0.42
Pregnancy rate (%)	26 (51/199)	28 (10/36)	0.79

Implantation rate was defined as the number of day 1 embryos with 2PN divided by the number of mature oocytes retrieved.

Fertilization rate was defined as the number of gestational sacs divided by the number of embryos transferred.

Pregnancy rate was defined as gestational sac visualized via ultrasound per recipient oocyte.
